# Comparative Effectiveness of Origami-Box-Folding and Outside-the-Box Knot-Tying Exercises in Laparoscopic Surgical Training: A Prospective Cohort Study

**DOI:** 10.3390/healthcare13212820

**Published:** 2025-11-06

**Authors:** Cristian-Valentin Toma, Adrian-Iustin Georgevici, Didina-Catalina Barbalata, George-Sabin Popescu, Ioana Gabriela Visan, George E. D. Petrescu, Cătălin Ovidiu Nechita, Daniel Liviu Bădescu, Cristian George Tieranu, Alexandru Ciudin, Viorel Jinga

**Affiliations:** 1 Department of Medical Simulation, Carol Davila University of Medicine and Pharmacy, 020021 Bucharest, Romania; cristian.toma@umfcd.ro (C.-V.T.); ioana.visan@umfcd.ro (I.G.V.); george.petrescu@umfcd.ro (G.E.D.P.); ovidiu-catalin.nechita@drd.umfcd.ro (C.O.N.); daniel.badescu@umfcd.ro (D.L.B.); cristian.tieranu@umfcd.ro (C.G.T.); viorel.jinga@umfcd.ro (V.J.); 2 Department of Urology, Prof. Dr. Theodor Burghele Clinical Hospital, 061344 Bucharest, Romania; 3 Department of Anesthesiology and Intensive Care Medicine, Katholisches Klinikum, St. Josef Hospital, Ruhr University, 44791 Bochum, Germany; igeorgevici@outlook.com; 4 Department of Obstetrics-Gynecology, Elias University Emergency Hospital, 011461 Bucharest, Romania; 5 Department of Neurosurgery, Bagdasar-Arseni Clinical Emergency Hospital, 041915 Bucharest, Romania; 6 Department of Gastroenterology, Elias University Emergency Hospital, 011461 Bucharest, Romania; 7 Department of Urology, Hospital Universitari de Mollet, Universidad de Barcelona, 08100 Barcelona, Spain; alexciudin@gmail.com

**Keywords:** simulation-based training, laparoscopic surgery, Fundamentals of Laparoscopic Surgery (FLS), Origami Box Folding Exercise (OBFE), Outside the Box Knot Tying Exercise (OBTKE)

## Abstract

**Background/Objectives**: Minimally invasive surgical techniques require precise psychomotor skills distinct from those used in traditional surgery. Simulation-based training is essential for skill acquisition without patient risk. This study compared two prevalent training methodologies: the Origami-Box-Folding Exercise (OBFE) and Outside-the-Box Knot-Tying Exercise (OBTKE). **Methods**: In this prospective cohort study, 84 surgical residents (34 OBFE, 50 OBTKE) from General Surgery, Obstetrics–Gynecology, and Urology underwent pre- and post-intervention assessments. Performance metrics included completion times for surgical and square knots, out-of-visual-field instrument instances, needle drops, tissue lesions, and self-assessment via 5-point Likert scales. Behavioral Observation Research Interactive Software quantified performance objectively. Data were analyzed using paired Wilcoxon signed-rank tests for within-group comparisons and Wilcoxon rank-sum tests for between-group differences. **Results**: Both methodologies significantly improved surgical knot-tying performance. Surgical knot completion time decreased by 316.65 s (OBFE) and 360 s (OBTKE) with no significant between-group difference (*p* = 0.96). For square knots, OBFE exhibited significantly greater improvement with a 278 s reduction versus 169 s for OBTKE (*p* = 0.02). Technical errors decreased similarly in both groups. OBFE showed greater improvement in self-rated surgical knot knowledge (*p* = 0.03) and larger effect sizes for self-assessment measures (0.84–0.87 vs. 0.77–0.85). **Conclusions**: Both OBFE and OBTKE effectively improve laparoscopic skills in surgical residents. OBFE is particularly beneficial for square knot efficiency and self-rated knowledge enhancement, while OBTKE focuses on targeted knot-tying training. These findings support the implementation of both methodologies in surgical education, potentially in sequence—OBFE for foundational skills and OBTKE for advanced refinement.

## 1. Introduction

Minimally invasive surgical techniques have transformed modern surgery by reducing postoperative pain, shortening hospital stays, and improving cosmetic outcomes. Laparoscopic surgery demands precise psychomotor skills and hand–eye coordination that differ from those involved in open surgery [1,2]. Thus, these skills pose substantial learning challenges for trainees, necessitating structured educational methods beyond traditional apprenticeships [3,4].

Medical simulation is essential for surgical education, ensuring a safe space for skill acquisition without risking patient safety [5]. While they are commonly incorporated into surgical curricula, the optimal methods for teaching laparoscopic skills remain under debate [4,6]. The Fundamentals of Laparoscopic Surgery (FLS) program recognizes box simulations, yet the evidence in favor of particular exercises in this context is still developing [7,8].

The aim of this study was to test two novel laparoscopic training methodologies: the Origami-Box-Folding Exercise (OBFE) and Outside-the-Box Knot-Tying-Exercise (OBTKE). OBFE involves folding an origami paper inside the laparoscopic training box. Therefore, through Japanese papercraft principles, trainees can improve hand–eye coordination, depth perception, and instrument handling dexterity [3,7,8,9]. OBTKE focuses on knot-tying skills outside the simulator. This allows for direct visualization of the knot-tying process, thus ensuring that novices learn how to perform the knots with the long rigid instruments without the constraints of laparoscopic surgery. As far as we are concerned, this methodology has not been previously implemented but is similar to the transparent laparoscopic box trainer approach developed by Rodrigues et al. [10].

Both methods address distinct aspects of the complex skill set required for minimally invasive surgery. In this study, the aim is to compare the effectiveness of these training methodologies in developing abilities in laparoscopic knot-tying, focusing on both objective and subjective performance metrics.

## 2. Materials and Methods

### 2.1. Study Design and Participants

This prospective study compares the laparoscopic knot-tying skills of 84 surgical residents, from General Surgery, Obstetrics–Gynecology, and Urology specialties trained using two different simulation methodologies: the Origami-Box-Folding Exercise (OBFE) and Outside-the-Box Knot-Tying Exercise (OBTKE).

Participants were recruited via announcements distributed in social media groups targeted towards local communities of surgical residents. An online registration form was provided for interested individuals in order to check eligibility and select availability windows. In order to be eligible, participants had to be enrolled in an accredited residency program in General Surgery (6-year program), Obstetrics–Gynecology (5-year program), or Urology (5-year program) at a teaching hospital in Bucharest. Due to logistical constraints in aligning participants’ availability with pre-scheduled dates for each training methodology, constrained randomization was used to assign participants to cohorts. All participants provided informed consent for GDPR compliance. In order to ensure the anonymity of the data, each participant was assigned a randomly generated numerical code, thus facilitating the blind processing of the data.

### 2.2. Training Methodologies

In both cohorts, participants attended one session of the workshop. Each session consisted of a pre-intervention assessment of laparoscopic knot-tying abilities, followed by one of the methodologies (OBFE vs. OBTKE) and a post-intervention assessment. Both pre- and post-intervention performances were recorded and further analyzed using Behavioral Observation Research Interactive Software (BORIS).

**Figure 1:**
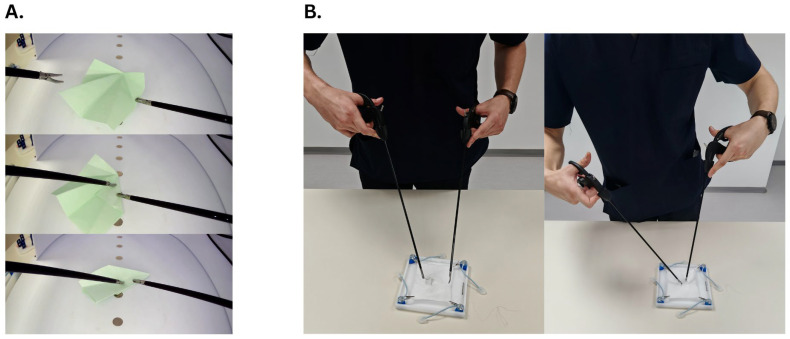
(**A**) Trainee performing OBFE task. (**B**) Trainee performing OBTKE task.

OBFE is performed using a laparoscopic training dry box where the trainee manipulates a single sheet of 7 × 7 cm origami paper using standard laparoscopic instruments. During the exercise, the trainee folds the paper into a paper plane shape (Figure 1A) following a previously provided step-by-step guide. The procedure requires precision in grasping, manipulating, and positioning the paper through the restricted movement of laparoscopic instruments.

OBTKE involves performing surgical and square knot-tying using laparoscopic instruments outside the training box (Figure 1B). The exercise allows direct observation of the procedure, providing a form of visual feedback that is often lacking in traditional laparoscopic training methods. Therefore, the trainee is able to develop the necessary depth perception when manipulating the laparoscopic instruments using three-dimensional vision prior to transitioning to the laparoscopic training box where the technique is facilitated by a camera.

### 2.3. Assessment Methods

A team of faculty members specialized in General Surgery, Obstetrics–Gynecology, and Urology assessed the knot-tying evaluated trainees’ performance using BORIS. Performance metrics included time to complete surgical and square knots (seconds), instances of instruments outside the visual field, needle drops, and tissue lesions. Theoretical knowledge was assessed via standardized tests. Self-assessment utilized 5-point Likert scales for perceived skill level, theoretical knowledge, and procedural confidence. Similarly, participants assessed the impact of the workshop on knowledge and dexterity acquisition. Demographic data included age, gender, residency year, specialty, previous laparoscopic box simulation and laparoscopic surgery experience, number of procedures performed, and previous surgical roles.

**Figure 2:**
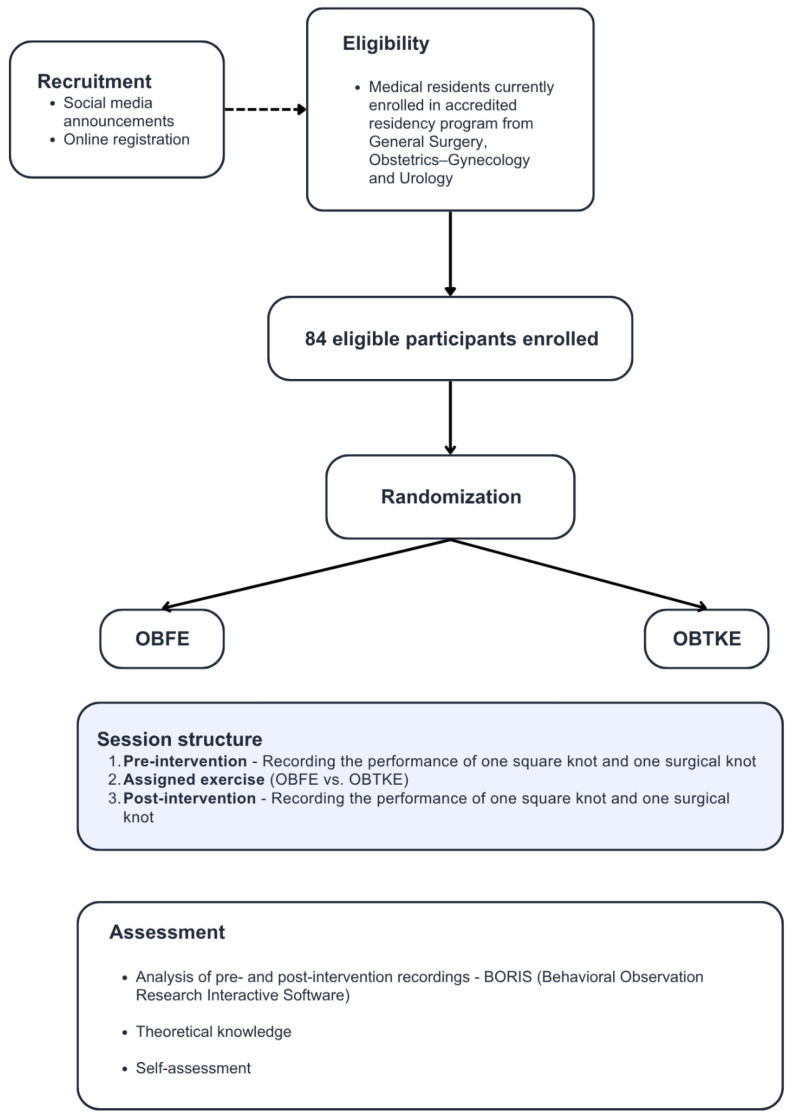
Flowchart depicting the course of the study.

Figure 2 summarizes the overall study design, including the recruitment eligibility screening, enrolment, practical session structure, and assessment.

### 2.4. Statistical Analysis

Demographic data were analyzed descriptively: continuous variables as medians (IQR: 25th, 75th percentile) and categorical variables as counts (percentages). Median differences between pre- and post-intervention measurements were calculated using interquartile ranges. Values with an absolute maximum of 25th or 75th percentile > 10 were rounded to the nearest integer; smaller values were reported to two decimal places. For categorical data, Pearson’s Chi-squared tests were applied for adequate expected counts, and Fisher’s exact tests were applied for low counts. Training effectiveness was evaluated with paired Wilcoxon signed-rank tests for pre- and post-intervention measurements of each module. *p*-values were adjusted using the Benjamini–Hochberg procedure to control the false discovery rate. Effect sizes (ESs) were calculated as ES = |qnorm(*p*-value/2)|/√sample size. Between-group comparisons used Wilcoxon rank-sum tests on difference scores (post-intervention minus pre-intervention) for each outcome measure. Statistical significance was set at *p* < 0.05. Data analysis was performed using R version 4.4.2.

## 3. Results

A total of 84 surgical residents participated in this study, with 34 assigned to the Origami Box-Folding-Exercise (OBFE) group and 50 to the Outside-the-Box Knot-Tying Exercise (OBTKE) group. As shown in Table 1, participants had similar median ages (27 years in OBFE vs. 26 years in OBTKE) and residency years (median 2 in OBFE vs. 1 in OBTKE), with no significant differences between groups.

Further demographic data from Table 1 shows that previous surgical experience varied between groups, with OBTKE participants reporting a higher median number of performed surgeries (35 vs. 15, *p* = 0.033), though this difference was attenuated after false discovery rate correction (q = 0.073). Few participants had previous experience with laparoscopic box simulation training (26% in OBFE vs. 40% in OBTKE), but most of them reported prior laparoscopic surgery experience (85% in OBFE vs. 96% in OBTKE), predominantly serving in camera operator roles (82% in OBFE vs. 66% in OBTKE).

Table 2 presents the distribution of participants across specialties: General Surgery (46%, n = 39), Obstetrics–Gynecology (27%, n = 23), and Urology (26%, n = 22). The same table shows that the majority (52%, n = 44) were first-year residents, followed by second-year residents (31%, n = 26), with the remaining participants distributed across years 3–5.

As detailed in Table 3 and Figure S1, both training methodologies produced substantial improvements in surgical knot-tying performance. Surgical knot completion time decreased by a median 316.65 s in the OBFE group and 360 s in the OBTKE group, with no significant difference between approaches (*p* = 0.96). However, for square knot performance, the OBFE group demonstrated significantly greater improvement with a median reduction of 278 s compared to 169 s in the OBTKE group (*p* = 0.02).

Performance quality metrics including time with instruments outside the visual field, instances outside the field, needle drops, and tissue lesions improved in both groups without significant between-group differences. Both interventions effectively reduced technical errors and enhanced procedural efficiency, with median reductions in tissue lesions of 1.00 for both surgical and square knots across both groups. The *p* value columns indicate that within-group improvements were statistically significant (*p* < 0.01) for most measures in both training methodologies. Theoretical evaluation scores showed consistent improvement (median increase of 1.00 point) across both groups.

Participants in both groups reported substantial improvements in self-assessment measures (Table 4). The OBFE group demonstrated significantly greater improvement in self-rated surgical knot knowledge (median increase of 2.00 vs. 1.00 points, *p* = 0.03). Self-confidence in performing surgical and square knots improved similarly in both groups (median increases of 2.00 points), indicating enhanced procedural comfort regardless of training methodology.

**Figure 3:**
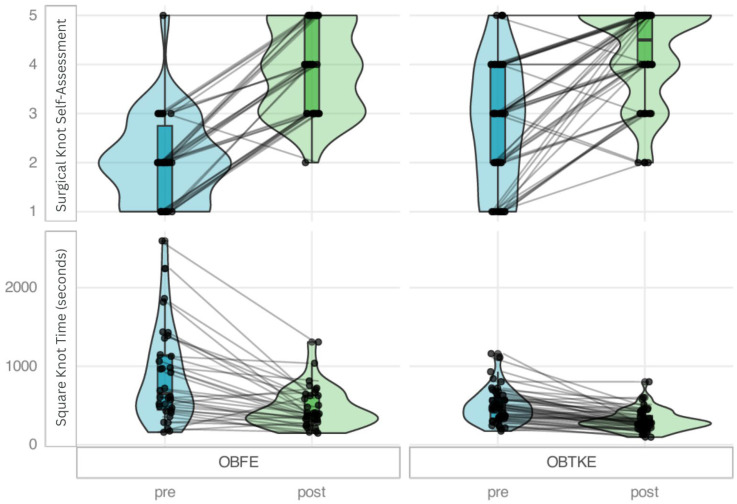
Metrics with the highest differences across training modules between pre- and post-intervention assessments per participant. For knot time, lower values, in seconds, indicate better performance (faster). For self-rated surgical knot knowledge (Likert scale), higher values show better self-assessment.

The effect sizes demonstrated substantial learning effects in both training modules. For self-assessment measures, OBFE consistently showed slightly larger effect sizes (0.84–0.87) compared to OBTKE (0.77–0.85) (Figure 3). For performance metrics, the pattern was mixed, with OBTKE showing larger effect sizes for some measures (e.g., square knot time: OBTKE = 0.82 vs. OBFE = 0.74) and OBFE showing larger effects for others (e.g., surgical knot tissue lesions: OBFE = 0.62 vs. OBTKE = 0.48).

After the training session, self-reported baseline assessments showed higher ratings for the acquisition of knowledge (*p* = 0.028) and dexterity (*p* = 0.006, q = 0.027) in the OBTKE group compared to the OBFE group.

Although participants in the OBTKE group had greater previous experience both in box simulation training (OBTKE = 40% vs. OBFE = 26%) and laparoscopic surgery (OBTKE = 96% vs. OBFE = 85%), this did not significantly impact their performance.

Despite some variation in specific outcomes, both training methodologies proved effective at improving technical skills and confidence in minimally invasive surgical techniques.

## 4. Discussion

Surgical residency programs are demanding, requiring trainees to acquire clinical knowledge and complex surgical skills while managing long work hours. In addition, the limited hands-on experience at the start of residents’ careers and the growing emphasis on patient safety and intraoperative efficiency make simulation-based training methodologies vital [11]. Despite the widespread implementation of standardized laparoscopic training programs, such as FLS [12], non-traditional training methodologies keep emerging. Alternative training methods such as video games [13,14,15], virtual reality [16,17], hand-made laparoscopic boxes [18,19], or origami paper folding [3,7,8,9] aim to promote laparoscopic skill acquisition in a manner that is both engaging and resource-efficient. These methods enhance structured training programs alongside traditional clinical education [20,21].

The Origami-Box-Folding Exercise (OBFE) and Outside-the-Box Knot-Tying Exercise (OBTKE) methodologies improve laparoscopic skills in surgical residents, each with distinct advantages. The OBFE emphasizes paper manipulation in confined spaces, fostering transferable skills beyond the immediate context [3]. OBTKE focuses on performing knots using laparoscopic instruments but without the constraints of two-dimensional vision, loss of depth perception, and limited freedom of movement. Mastering the knot-tying technique before working in the laparoscopic box allows the aforementioned limitations to be seamlessly overcome [10].

Both training methodologies reduced technical errors; however, their impact on skills varied due to training focus. The OBFE particularly boosts square knot efficiency and self-rated knowledge, while both techniques enhance surgical knot performance and decrease technical errors. Conversely, the OBTKE’s focus on knot-tying may explain its similar performance to OBFE in terms of surgical knot completion time, despite its narrower focus.

Performance metrics (completion time, instances outside the visual field, needle drops, tissue lesions) align with Gazis et al. and Laski et al. for trainee assessment [4,5]. These measures provide insights into the effectiveness of training methodologies beyond self-assessment. Dawe et al.’s systematic review highlights the significance of these metrics, revealing that participants proficient in simulation-based training outperformed in patient-based settings across various performance parameters [22].

Larger effect sizes for self-assessment measures in the OBFE group suggest enhanced confidence in laparoscopic skills, aligning with the literature on spatial awareness and instrument handling [3,9]. However, participants with prior involvement in laparoscopic surgery-related activities seemed to provide more critical assessments regarding their performance and the intervention’s impact on their abilities.

Our findings support the complementarity of these training approaches. The OBTKE ensures knot-tying proficiency [10], whilst OBFE improves general laparoscopic skills and spatial awareness [3,7,8,9]. Papanikolaou et al. demonstrated that adding box trainers to traditional surgical training significantly enhanced residents’ performance in movements, operative time, and overall surgical skills, with lasting improvements [23].

Both methods significantly improved procedural confidence, corroborating Raj et al.’s finding that box trainers develop essential surgical skills [17] and Schwab et al.’s focus on competency-based education and mastery learning as vital in effective surgical education programs [11].

## 5. Limitations

The present study has several limitations that should be acknowledged. Firstly, it should be noted that individual factors such as age, dexterity, and prior fine motor experience impact performance and skill acquisition [16,21]. Secondly, the study assessed immediate performance improvements but did not investigate the retention of skills over time or clinical transferability. Therefore, the long-term impact of these interventions remains unclear. The results could be strengthened by evaluating a larger sample of surgical residents over multiple training sessions, thus ensuring sustainable retention of skills. Further research should focus on the analysis of results between subgroups and developing training methodologies targeted towards the needs of different stages of surgical residency training and each surgical specialty.

As previously mentioned in the Materials and Methods section, the randomization process of the cohorts was hindered by logistical constraints, leading to slightly heterogeneous and unequal study groups. We acknowledge that the imbalance between the baseline skills of the participants may weaken the study design and reduce the strength of the results. However, the heterogeneity reflects real-life training environments, where residents often begin with different levels of prior experience in the surgical field. Thus, the study offers insights into how the training program performs under practical conditions.

The study focused on quantifiable performance metrics, not taking into account cognitive and psychological factors that could influence technical proficiency. Elements such as decision-making abilities, environmental stress factors, or mental workload could affect the surgical outcomes. Consequently, the results may not fully reflect intraoperative proficiency, as surgical competence extends beyond technical skills.

## 6. Conclusions

Our findings prove that OBFE and OBTKE are feasible alternative methodologies for basic skills laparoscopic training, ensuring the development of fine motor control and spatial awareness. Future research should emphasize long-term skill retention, clinical application, and optimal training sequencing in surgical curricula. Moreover, exploring cost-effectiveness will inform resource allocation in surgical education.

## Figures and Tables

**Table 1:** Demographic characteristics.

	Overall N = 84	OBFE N = 34	OBTKE N = 50	*p*-Value	q-Value	Test
Age	26 (25, 27)	27 (25, 28)	26 (25, 27)	0.12	0.20	W
Residency year	1 (1, 2)	2 (1, 2)	1 (1, 2)	0.19	0.23	W
Previous laparoscopic box simulation experience (yes)	29/84 (35%)	9/34 (26%)	20/50 (40%)	0.20	0.23	chi
Laparoscopic surgery experience (yes)	77/84 (92%)	29/34 (85%)	48/50 (96%)	0.11	0.20	F
*Camera*	61 (73%)	28 (82%)	33 (66%)			
*Secondary role*	12 (14%)	3 (8.8%)	9 (18%)			
*Observatory*	11 (13%)	3 (8.8%)	8 (16%)			
Knowledge acquisition (Likert scale)	5 (4, 5)	4 (4, 5)	5 (4, 5)	0.03	0.09	W
Dexterity acquisition (Likert scale)	5 (4, 5)	4 (4, 5)	5 (4, 5)	<0.01	0.05	W

*Overall, OBFE, OBTKE: median (Q1, Q3); n/N (%); n (%). Q-value: false discovery rate correction for multiple testing. Test: W, Wilcoxon rank-sum test; chi, Pearson’s Chi-squared test; F, Fisher’s exact test.*

**Table 2:** Surgical specialty in residency by training module.

	General Surgery	Obstetrics and Gynecology	Urology	Total	*p*-Value
Residency year					0.56
I	21	11	12	44	
II	13	6	7	26	
III	3	3	0	6	
IV	1	2	3	6	
V	1	1	0	2	
**Total**	39	23	22	84	

**Table 3:** Objective assessment differences between OBFE and OBTKE modules.

	Post-Pre OBFE	Post-Pre OBTKE	Effect Size OBFE	Effect Size OBTKE	*p*-Value OBFE	*p*-Value OBTKE	*p*-Value Between Modules
	Square Knot						
Completion time (s)	−278 (−763, 36)	−169 (−332, −23)	0.74	0.82	0.01	<0.01	0.02
Instances outside field	0 (−1, 0)	0 (−2, 0)	0.37	0.52	0.03	<0.01	0.56
Time outside field (s)	0 (−7.7, 0)	0 (−3.9, 0)	0.53	0.45	<0.01	<0.01	0.53
Tissue lesions	−1 (−2, 0)	−1 (−2, 0)	0.52	0.63	<0.01	<0.01	0.81
Needle drops	0 (−1, 0)	−1 (−1, 0)	0.30	0.60	0.08	<0.01	0.28
	Surgical Knot						
Completion time (s)	−316 (−775, −1)	−360 (−714, −42)	0.79	0.85	<0.01	<0.01	0.96
Instances outside field	0 (−1, 0)	0 (−2, 0)	0.26	0.47	0.14	<0.01	0.30
Time outside field (s)	0 (−13.68, 0)	0 (−8.44, 0)	0.55	0.55	<0.01	<0.01	0.70
Tissue lesions	−1 (−2, 0)	0 (−1, 0)	0.62	0.48	0.01	<0.01	0.32
Needle drops	0 (−2, 0)	0 (−1, 0)	0.54	0.49	<0.01	<0.01	0.66
Theory test score	1 (0, 2)	1 (0, 2)	0.75	0.77	<0.01	<0.01	0.32

*The columns show the median difference (post-intervention minus pre-intervention) in knot performance and theoretical knowledge, effect sizes (ESs) and level of significance (*p*-values). Negative time values indicate performance improvement (reduced completion time). The ESs indicate the magnitude of the training effect, with larger values indicating more substantial improvements. The *p*-values for OBFE and OBTKE show the differences (using Wilcoxon paired rank-sum tests) between post- and pre-intervention within training modules. The last *p*-value shows the differences across training modules.*

**Table 4:** Self-assessment differences between OBFE and OBTKE modules.

	Post-Pre OBFE	Post-Pre OBTKE	Effect Size OBFE	Effect Size OBTKE	*p*-Value OBFE	*p*-Value OBTKE	*p*-Value Between Modules
	Square Knot						
Self-confidence	2 (1, 3)	2 (1, 3)	0.86	0.84	<0.01	<0.01	0.60
Self-rated knot-tying skills	2 (1, 3)	2 (1, 3)	0.87	0.85	<0.01	<0.01	0.25
Self-rated knot-tying knowledge	2 (1, 3)	2 (1, 3)	0.87	0.84	<0.01	<0.01	0.17
	Surgical Knot						
Self-confidence	2 (1, 3)	2 (1, 3)	0.84	0.82	<0.01	<0.01	0.90
Self-rated knot-tying skills	2 (1, 2)	2 (1, 3)	0.87	0.82	<0.01	<0.01	0.56
Self-rated knot-tying knowledge	2 (1, 3)	1 (0, 3)	0.86	0.77	<0.01	<0.01	0.03

*The columns show the median score improvement (post-intervention minus pre-intervention), effect sizes (ESs), and level of significance (p-values). The ESs indicate the magnitude of the training effect, with larger values indicating more substantial improvements. The p-values for OBFE and OBTKE show the differences (using Wilcoxon paired rank-sum tests) between post- and pre-intervention within training modules. The last p-value shows the differences across training modules.*

## Data Availability

The raw data supporting the conclusions of this article will be made available by the authors on request from the corresponding author due to ethical reasons.

## Supplementary Materials

The following supporting information can be downloaded at https://www.mdpi.com/article/10.3390/healthcare13212820/s1, Figure S1: Additional pre- and post-intervention assessments per participant and training module.
