# Supplementary material for: Comparative Effectiveness of Origami-Box-Folding and Outside-the-Box Knot-Tying Exercises in Laparoscopic Surgical Training: A Prospective Cohort Study
Source: Healthcare (Basel). 2025 Nov 6;13(21):2820. doi: 10.3390/healthcare13212820 (PMC12610036; doi:10.3390/healthcare13212820)
Supplement: Supplementary file 1 [file healthcare-13-02820-s001.zip › healthcare-3788171-supplementary.pdf]

Supplementary Materials

Figure S1: Additional pre- and post-intervention assessments per participant and training module

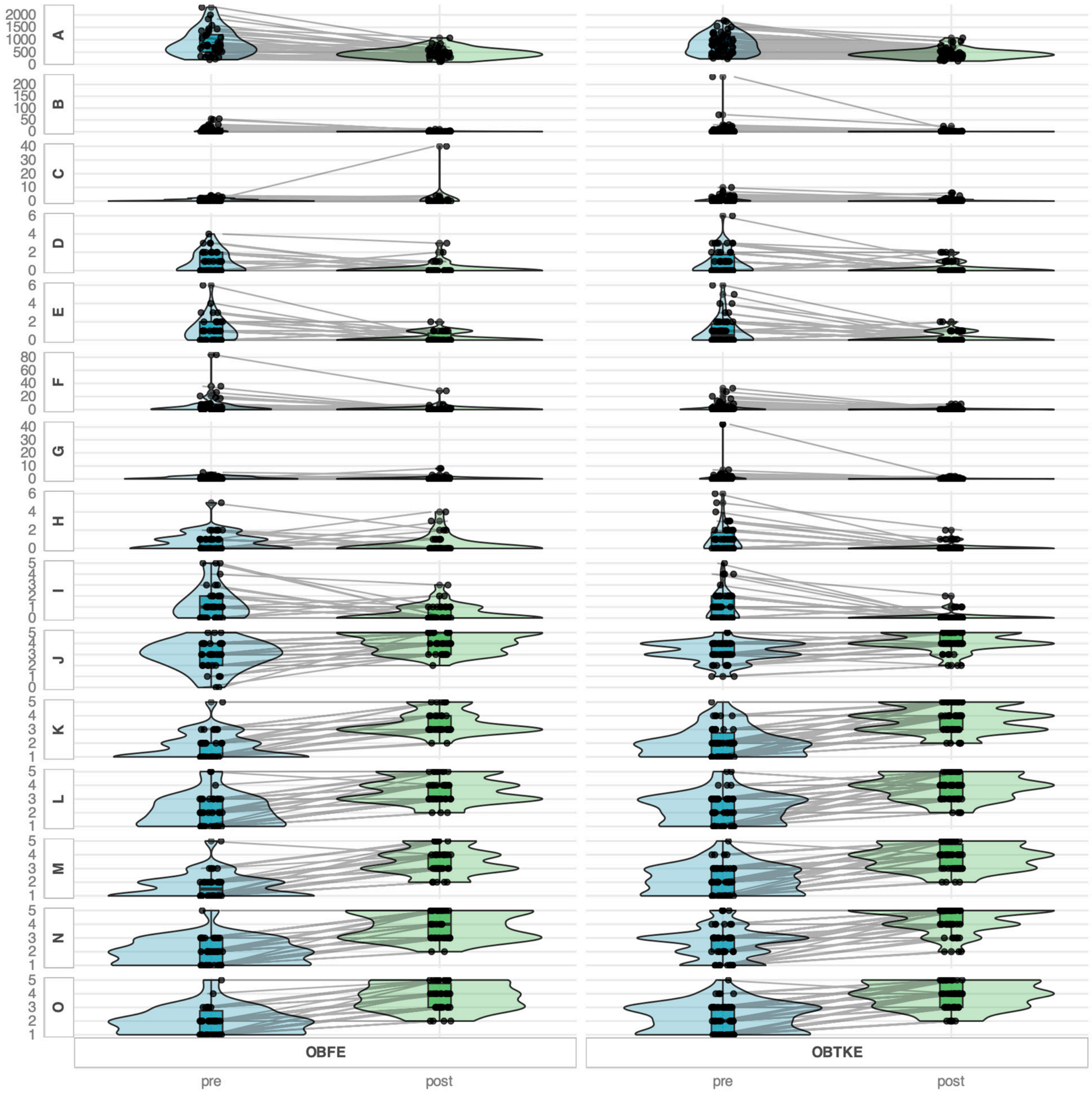

A Surgical Knot Time; B Surgical Knot: Time Outside Field; C Surgical Knot: Instances Outside Field; D Surgical Knot: Needle Drops; E Surgical Knot: Tissue Lesions; F Square Knot: Time Outside Field; G Square Knot: Instances Outside Field; H Square Knot: Needle Drops; I Square Knot: Tissue Lesions; J Theory Test Score; K Self-rated Surgical Knot Skills; L Self-confidence in Surgical Knot; M Self-rated Square Knot Skills N Self-rated Square Knot Knowledge O Self-confidence Square Knot.
